# Neighborhood Environment and Cardiometabolic Disease in Individuals Aging With Physical Disability

**DOI:** 10.1093/geroni/igab046.1837

**Published:** 2021-12-17

**Authors:** Anam Khan, Paul Lin, Neil Kamdar, Elham Mahmoudi, Philippa Clarke

**Affiliations:** 1 University of Michigan School of Public Health, Ann Arbor, Michigan, United States; 2 University of Michigan, Ann Arbor, Michigan, United States; 3 University of Michigan, Commerce Township, Michigan, United States; 4 Institute for Social Research, Ann Arbor, Michigan, United States

## Abstract

The environment may be particularly important for facilitating participation and health for individuals aging with physical disability. However, little is known about which features of the neighborhood are particularly pertinent for this population. This study aims to address this gap by examining the type(s) of neighborhood environments associated with cardiometabolic disease. We identified ~26,000 individuals with a diagnosis of physical disability using a national private health insurance claims database in the U.S. Geocoded information for individuals was used to assign them to features of their neighborhood from the National Neighborhood Data Archive. An adapted typology was used to classify neighborhoods into the following based on density of health-promoting and harming features: 1) High health-promoting/harming (service-dense), 2) High health-promoting/low harming, 3) Low health-promoting/high harming, 4) Low health-promoting/harming, and 5) Average. We used time-varying Cox models to estimate adjusted hazard ratios (HR) and 95% confidence intervals (CI) for time-to incident cardiometabolic conditions. High neighborhood-level affluence, and low disadvantage scores characterized service-dense neighborhoods. They had more than 2x higher density of health-promoting resources (e.g., transit) compared to other neighborhood types. Individuals residing in service-dense neighborhoods had an 8% lower risk of any cardiometabolic disease (HR 0.92, 95% CI: 0.85-0.99) compared to those in average neighborhoods. Similar effects were observed for Hypertension and Type 2 Diabetes, with effects most pronounced for the latter (HR 0.82, 95% CI: 0.71-0.94). For individuals aging with physical disabilities, service-dense neighborhoods may be protective against cardiometabolic morbidity. Findings can inform community design that support cardiometabolic health in this population.

